# Amyloid Self-Assembly of Lysozyme in Self-Crowded Conditions: The Formation of a Protein Oligomer Hydrogel

**DOI:** 10.1021/acs.biomac.0c01652

**Published:** 2021-02-18

**Authors:** Sara Catalini, Diego R. Perinelli, Paola Sassi, Lucia Comez, Giovanni F. Palmieri, Assunta Morresi, Giulia Bonacucina, Paolo Foggi, Stefania Pucciarelli, Marco Paolantoni

**Affiliations:** † European Laboratory for Non-Linear Spectroscopy (LENS), University of Florence, 50019 Sesto Fiorentino, Italy; ‡ School of Pharmacy, University of Camerino, 62032 Camerino, Italy; § Department of Chemistry, Biology and Biotechnology, University of Perugia, 06123 Perugia, Italy; ∥ IOM-CNR c/o Department of Physics and Geology, University of Perugia, 060123 Perugia, Italy; ⊥ School of Pharmacy, University of Camerino, 62032 Camerino, Italy; # National Metrological Research Institute (INRIM), Strada delle Cacce 91, 10135 Torino, Italy; ○ School of Biosciences and Veterinary Medicine, University of Camerino, 62032 Camerino, Italy

## Abstract

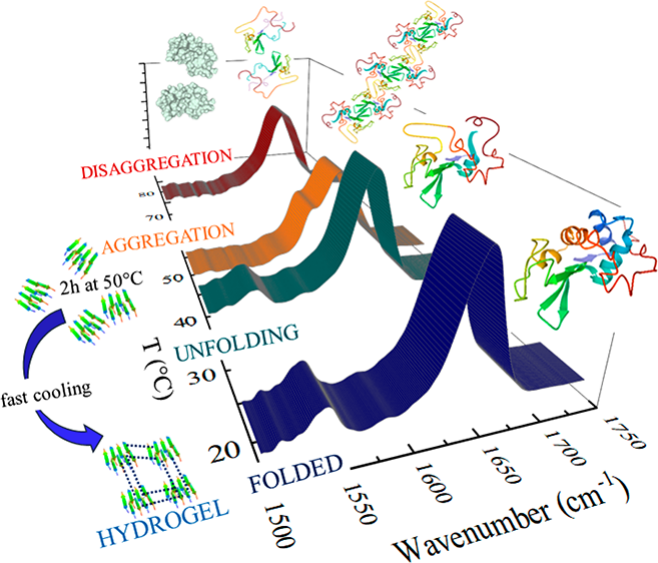

A method is designed to quickly form protein hydrogels, based on the self-assembly of highly concentrated lysozyme solutions in acidic conditions. Their properties can be easily modulated by selecting the curing temperature. Molecular insights on the gelation pathway, derived by in situ FTIR spectroscopy, are related to calorimetric and rheological results, providing a consistent picture on structure–property correlations. In these self-crowded samples, the thermal unfolding induces the rapid formation of amyloid aggregates, leading to temperature-dependent quasi-stationary levels of antiparallel cross β-sheet links, attributed to kinetically trapped oligomers. Upon subsequent cooling, thermoreversible hydrogels develop by the formation of interoligomer contacts. Through heating/cooling cycles, the starting solutions can be largely recovered back, due to oligomer-to-monomer dissociation and refolding. Overall, transparent protein hydrogels can be easily formed in self-crowding conditions and their properties explained, considering the formation of interconnected amyloid oligomers. This type of biomaterial might be relevant in different fields, along with analogous systems of a fibrillar nature more commonly considered.

## 1 Introduction

Formation of amyloid aggregates has been subjected to a huge number of investigations due to their implication in relevant diseases.^1−4^ The capability of giving rise to amyloid fibrils is a general property of non-native proteins, and populating unfolded states at high temperature represents a simple approach to induce their aggregation.^5−8^ This is generally an irreversible process,^9^ even if its reversibility has been observed.^10^ As a model protein, lysozyme (LYS) has received considerable attention due to its propensity to form *in vitro* amyloid fibrils.^11^ Incubation at high temperature and low pH represents the preferential route for their production,^2,11−17^ while at neutral or high pH, amorphous aggregation is favored.^18−21^ The LYS self-assembly at low pH has been deeply investigated, evidencing the formation of pathway-specific products.^22−24^ Two distinct fibril growth pathways are identified: one at high ionic strength involving the formation of oligomeric aggregates and another at low ionic strengths in which single filaments grow up by addition of monomers.^22,23^ Both mechanisms are amyloidogenic and lead to structurally differentiable fibrils, depending on the early stages of self-assembly.^23^ The growth of rigid fibrils and globular oligomers are independent; oligomers might transform in curvilinear fibrils or amorphous precipitates, but do not convert into stable rigid fibrils.^25^ The role of globular oligomers and curvilinear fibrils as off-pathway competitors with rigid fibrils has been recently confirmed.^26^ Oligomers are often considered as mainly responsible for cellular toxicity,^4,24−26^ but being transient species, their study is inherently challenging.^4^

In recent years, amyloid fibrils have received additional attention as functional components of novel biocompatible materials of relevance in biomedical and biotechnological applications.^27−30^ Examples of suitable biomaterials include tailored protein hydrogels constituted by a network of self-assembled nanofibrils in water.^28,30^ The formation of amyloid hydrogels within the intra- and extracellular medium might represent itself a possible mechanism of cell toxicity.^31,32^ Exploiting its propensity to form fibrillary networks, LYS has been employed to create different functional biomaterials, such as hydrogels as a scaffold for cell cultures,^33−35^ fibril networks with controllable morphologies,^36^ and nontoxic microgels suitable for drug delivery.^37^ Aggregation of globular protein is also of great interest in food technology. Indeed, the formation of aggregates with different morphologies and of tunable gels network might represent a useful way to improve food properties and preservation.^38−41^

Most investigations connected to human diseases or functional materials, have considered the formation of amyloid fibrils at low protein concentrations. Studies on LYS are usually performed at concentrations lower than 10 wt %, with typical values within 1–4 wt %.^13,22,23,32−36,42,43,44^ In these conditions, the growth of amyloid fibrils generally takes place at temporal scales of days and often is triggered by amyloidogenic fragments formed by hydrolytic processes.^36,43,44^ Less efforts have been devoted to study LYS unfolding and aggregation at higher concentrations, with protein contents larger than 10 wt % (∼100 mg/mL).^45−50^ Nevertheless, understanding the behavior of crowded samples is of relevance in cellular biology, industrial, and pharmaceutical fields.^45,51−56^ A broad range of pharmaceutical applications require formulations of very concentrated samples (>100 mg/mL), rising issues caused by protein aggregation.^48,51,52^ The need of methods to monitor in situ concentrated protein samples has clearly emerged.^48,51,57^ Studying proteins in crowded solutions is also helpful to explain their behavior within the cellular environment,^53,54,56^ where the total macromolecules concentration is extremely high (up to 400 mg/mL).^56,58^ At this level of crowding, excluded volume and viscosity are relevant factors in determining aggregation features. For instance, the acceleration of α-synuclein fibrillation induced by a crowding agent is ascribed to the excluded volume effects.^58^ These and other factors act even in the case of self-crowding,^59,60^ affecting the stability of the protein^61^ and its aggregation due to thermodynamic and kinetic reasons, which also might involve the water environment.^60^ Formation of (transient) clusters of native LYS is expected for concentrations larger than ∼100 mg/mL.^62^ LYS melting temperature is found to increase significantly with concentration in diluted regimes (up to ∼15 mg/mL) due to the excluded volume.^63^ Instead, at high protein content, a thermal destabilization upon self-crowding, attributed to enthalpic effects, is revealed.^47,50^ Yet, no changes in the secondary structure of LYS are evidenced within 2.5–300 mg/mL,^46^ but additional effects are expected if specific aggregates will form upon denaturation.^45^ It appears that a deep analysis of unfolding and (amyloid-like) association in self-crowded samples, rarely explored for this common model protein, might reveal novel insights of relevance in several areas, including cellular biology, medicine, biotechnology, and food sciences.

In this work, the thermal denaturation, aggregation and gelation occurring in a very concentrated LYS sample (∼240 mg/mL) are investigated in situ by means of FTIR spectroscopy, particularly sensitive to amyloid structures,^33−35,64^ and differential scanning calorimetry (DSC). Protocols are selected to form different protein hydrogels in reduced temporal scales (a few hours), monitoring in real-time the whole self-assembling process at different temperatures. A selected hydrogel is then investigated by noninvasive FTIR, DSC, and rheological experiments, providing a consistent interpretation of its properties, based on the formation of kinetically trapped amyloid oligomers.

## 2 Experimental Section

### 2.1 Materials

The lyophilized powder of hen egg-white lysozyme (Sigma-Aldrich, L6876) is dissolved without further purification in deuterium oxide (99.9 atom % D, Sigma-Aldrich) to obtain solutions with concentrations of 60, 120, and 240 of mg of solute/mL of solvent (here referred as mg/mL), corresponding to 5, 10, and 18 wt % and denoted as LYS60, LYS120, LYS240, respectively. The sample is left overnight to ensure total protein dissolution. The pD is then adjusted to 1.8, adding small amounts of 2 M deuterium chloride (DCl) to reach a pH meter reading of 1.4; as usually done, since deuterated water is employed, 0.4 units should be added to the pH meter reading.

### 2.2 FTIR Spectra

Infrared absorption measurements are collected using a FTIR Bruker spectrometer model Tensor27, equipped with a DTGS detector. The Opus 5.5 Bruker Optics software allowed the acquisition and the analysis of spectra. Transmission spectra are obtained employing a homemade cell equipped with CaF_2_ windows; the cell is positioned into a jacket whose temperature is controlled by circulating water through a Haake F6 thermostat. To monitor visually macroscopic changes, parallel thermal treatments are performed on sample aliquots placed into standard cuvettes. The spectra are acquired with a resolution of 2 cm^–1^ by averaging over 20 scans for each spectrum. To track the temperature and time dependence of the amide I peak position located at 1650 cm^–1^, the center of gravity of the band is determined at 20% from the maximum intensity with the corresponding Opus 5.5. routine.

### 2.3 Micro-DSC Thermograms

Microcalorimetry analyses are performed using a micro-DSC III (Setaram, France). First, 0.750 g of LYS dispersion at the three different concentrations (60, 120, and 240 mg/mL) are loaded inside a Hallostey calorimetric cell and analyzed using the following thermal program: isotherm at 20 °C for 20 min followed by a consecutive heating and cooling ramp from 20 to 80 °C at 1 °C/min. The same thermal program is used also to analyze the LYS gel, formed through the thermal treatment involving curing at 50 °C, as described below. The temperature and the enthalpy are calculated from the peak and the area of the transition using the tangent method.

### 2.4 Rheological Experiments

Viscoelastic properties of the LYS gel matrix is studied using a rotational rheometer (Kinexus, Malvern) equipped with a 20 mm plate geometry at a gap of 1 mm. The frequency sweep tests are performed at a shear stress of 0.5 Pa in the frequency range 0.01–10 Hz at temperatures of 15, 25, 35, and 50 °C. The temperature sweep test is conducted at a frequency of 1 Hz and a stress of 0.5 Pa between 15 and 70 °C at a rate of 1 °C/min. The time sweep test at 0.5 Pa and 1 Hz at two temperatures (50 and 25 °C) for 20 min is made and repeated for both temperatures two times consecutively.

## 3 Results and Discussion

### 3.1 Thermal Unfolding in a Self-Crowded Nonaggregating Solution (LYS120)

Before discussing the results obtained for the LYS240 sample, the FTIR analysis of the LYS120 solution is illustrated, as a reference sample that, albeit still very self-crowded, does not produce amyloid-type aggregates. The aim is to obtain the melting parameters of the protein in a concentrated mixture (>100 mg/mL) in the absence of aggregation and to validate a procedure, based on deuterium exchange experiments, to probe the protein thermal stability in the LYS240 sample for which the development of aggregates interferes with the classical FTIR spectral analysis.

**Figure 1:**
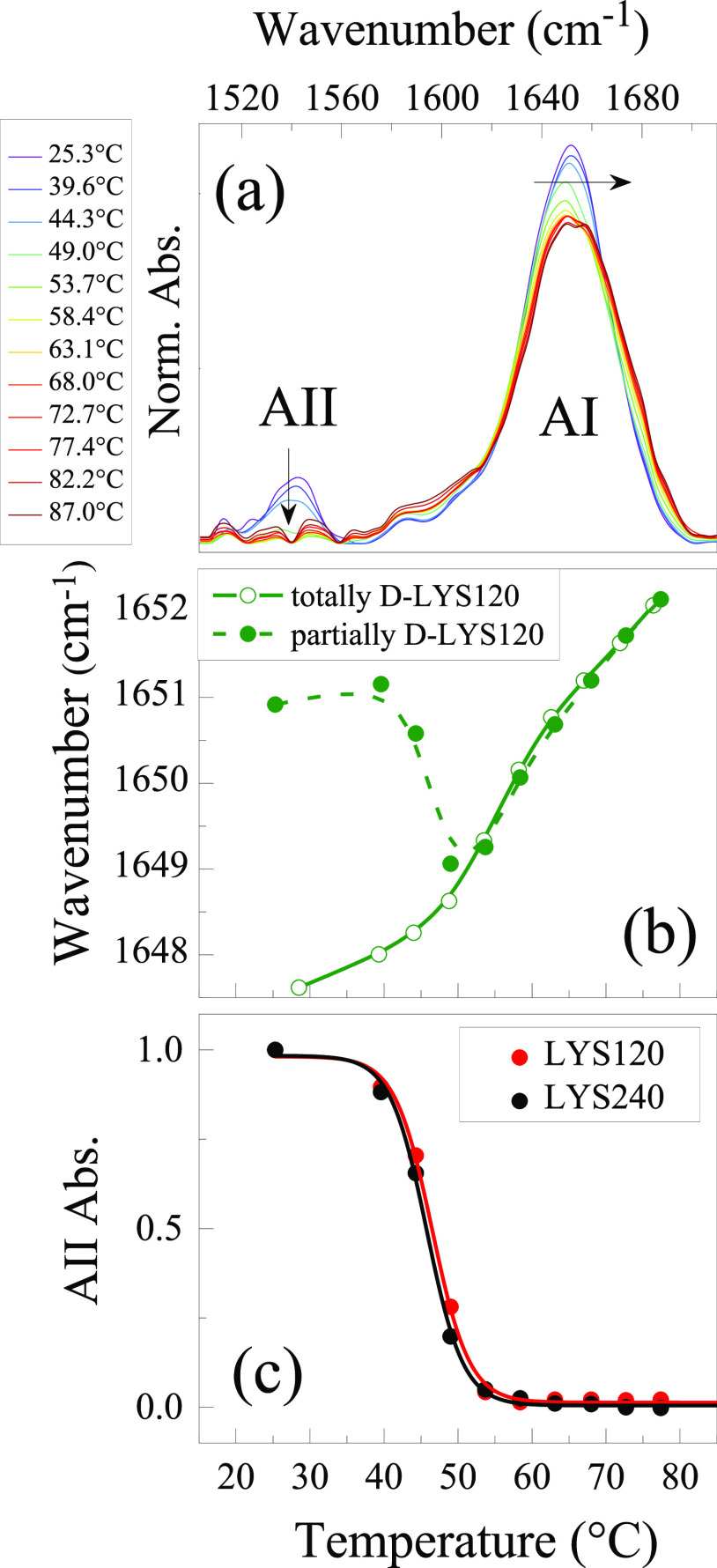
(a) FTIR spectra of the LYS120 solution recorded as a function of temperature in the amide I (AI) and amide II (AII) regions. (b) AI frequency shift of the partially and the totally deuterated LYS120 samples. (c) AII absorbance of partially deuterated LYS samples determined at different temperatures: LYS120 (red curve) and LYS240 (black curve) solutions.

The thermal unfolding of LYS120 is characterized by analyzing the amide I (AI) and amide II (AII) bands in the FTIR spectra recorded from 25 to 87 °C (Figure 1a).

Figure 1b shows that the AI peak position (green full circles) shifts to lower wavenumbers in the range 40–50 °C and then progressively moves to higher values. The red-shift is caused by the H/D exchange between the D_2_O solvent and the amide hydrogens localized in the core of the folded structure;^65,66^ this is also indicated by the parallel decrease of the AII band (Figure 1a), which is mainly ascribed to a N–H bending vibration. The full H/D exchange is reached at the exchange temperature *T*_ex_ of 50 °C (Figure 1c), a few degrees below the melting temperature *T*_m_ = 53 °C (see hereafter), as also observed in other cases.^64,65,67^ This fact has been related either to a change in the tertiary structure that precedes the global unfolding, giving rise to an intermediate with a native-like secondary structure or to conformational changes of more local and transient characters.^64,67^ Anyhow, it can be simply explained considering a cooperative two-state process, involving folded (F) and unfolded (U) species in thermal equilibrium.^64,65^ In fact, if the exchange of internal N–H groups becomes fast enough in the U state, as expected, even the presence of a small fraction of U species at *T* < *T*_m_ will lead to a rapid exchange of the total amount of protein. In these conditions, the signal depletion rate is expected to depend directly on the fraction of U species.^68−71^

The AI peak position for LYS120 totally exchanged after a 1 h incubation at 50 °C (Figure 1b: green empty circles) follows a pseudosigmoidal functional form, evidencing a melting region at around 50–60 °C and both pre- and postmelting linear domains; for *T* > *T*_ex_, the curves obtained with or without the pretreatment recover. Similar melting curves, previously observed for LYS solutions containing denaturing cosolvents (ethanol and DMSO), are interpreted based on a classical two-state picture, with a rather continuous thermal restructuring within the F and U states themselves.^65,72,73^ Based on such a model, thermodynamic parameters are extracted at different pH values, as described in the Supporting Information. Going from pH = 4.0 to 2.8 only leads to minor changes to *T*_m_, from 75 to 72 °C (Table S1), in line with other FTIR studies.^45^ Relevant effects are instead observed at pH = 1.8 when both the *T*_m_ and enthalpy change (Δ*H*_F–U_) decrease down to 53 °C and ∼70 kcal/mol, respectively. These results agree with those obtained using different approaches in a range of lower concentrations at pH = 2: *T*_m_ ∼ 52.5 (3 mg/mL),^63^*T*_m_ = 54.8 (∼20 mg/mL),^42^ and *T*_m_ = 55 (100 mg/mL).^48^

The *T*_m_ and Δ*H*_U–F_ obtained at pH = 1.8 are practically coincident with those (*T*_m_ = 51 °C and Δ*H*_U–F_ ∼ 70 kcal/mol) previously obtained for a LYS solution (120 mg/mL; pH = 3.0) in the presence of ethanol (18% mole fraction); yet, in that case, the development of a large amount of ordered β-sheet aggregates was observed in the 30–64 °C range^72^ and rapid gelation at the macroscopic level.^74^ Thus, even if at any given *T*, the fraction of U species, prone to aggregation, must be similar in the two environments, the self-assembly is strongly suppressed at pH = 1.8. This can be mainly ascribed to the effect of Coulombic repulsive interactions among heavily charged proteins (a net protein charge of 17–18 is predicted at the working pH);^75^ the role of ethanol in favoring aggregation might be also taken into account. From a methodological point of view, we notice that the estimated *T*_ex_ is 3 °C lower than *T*_m_, similar to what was found for LYS in other solvent conditions;^65^ this indicates that *T*_ex_ is a reasonable approximation of the melting temperature *T*_m_, as long as comparable FTIR acquisition procedures are considered.

### 3.2 Thermal Unfolding and Aggregation in Very Concentrated Conditions (LYS240)

**Figure 2:**
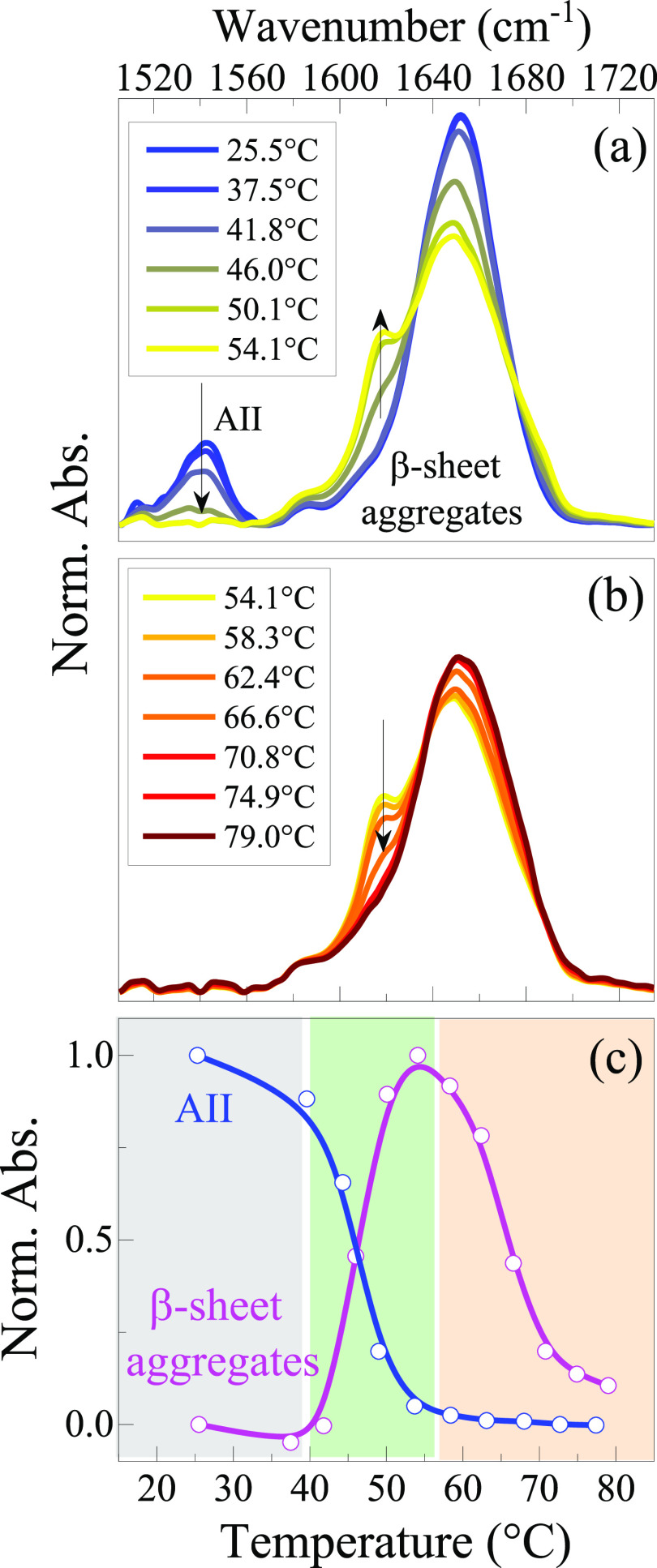
FTIR spectra in the amide I (AI) and amide II (AII) regions obtained for LYS240 in two temperature ranges: (a) from 25.5 to 54.1 °C and (b) from 54.1 to 79.0 °C. Panel (c) shows the temperature dependence of the aggregate signal absorbance at 1618 cm^–1^ together with the AII absorbance depletion resulting from the depicted spectra in panels a and b.

The same analysis previously performed for the LYS120 solution is conducted for the LYS240 sample. The corresponding thermal evolution of the AI and AII bands is shown in Figure 2. In this crowded sample, within the range 30–55 °C, the protein self-association is found to occur, as testified by the increase of the component peaked at ∼1618 cm^–1^, which is diagnostic for aggregates with amyloid structure.^23,24,72,73,76^ More specifically, the concomitant increase of an additional small component at 1690 cm^–1^ indicates the formation of interprotein antiparallel β-sheets.^33,34,43,64^ Considering the rapid growth (a few minutes) during temperature increasing, these bands relate to the first stages of self-association and must be attributed to small aggregates, in agreement with previous interpretations.^72,73,76^ In fact, the formation of amyloid fibril or even protofibrils at low pH requires a longer incubation period going from several hours to days, depending on the experimental conditions.^13,22,23,25^ Based on the mechanism of amyloid growth proposed by Hill et al.,^13^ in more diluted conditions, LYS oligomers are found to rapidly develop at 50 °C without nucleation barrier during the lag phase that precedes the protofibril nucleation step. These oligomers are considered metastable species that might further originate oligomer precipitates (O-Ppt) or curvilinear fibrils (CFs), but not thermodynamically stable rigid fibrils (RF).^25^ As such, we will refer to the amyloid aggregates identified by the two signals at 1618 and 1690 cm^–1^ as amyloid oligomers. Even if the presence of CFs cannot be completely excluded, their formation seems very unlikely considering the fast development of the amyloid aggregates we have observed.^25^ This assignment is supported by the conclusions of Zou et al.^43^ who specifically attributed the two spectral components at around 1618 and 1690 cm^–1^ to antiparallel β-sheet configurations of LYS oligomers. Differently, amyloid fibrils formed at 62 °C after long incubation periods (tens of hours) are expected to possess parallel β-sheet arrangements.^43^

Because of the occurrence of aggregation, it is challenging to monitor the unfolding process in this concentrated sample using the AI signal. On the other hand, we can easily follow the intensity decrease of the AII band at 1540 cm^–1^ due to the H/D exchange. As previously mentioned, the overall exchanging rate depends on the fraction of U species, thereby the intensity reduction of the AII band can be related to the position of the F ↔ U equilibrium.^65,68−71^Figure 1c shows similar depletion trends for the two LYS120 and LYS240 samples, leading to the same exchange temperature (*T*_ex_ ∼ 50 °C). This suggests that, at high concentrations, the melting process is basically independent of the protein content, even when strong self-association occurs. An analysis of the exchange kinetic at 45 and 50 °C further supports the notion that the F ↔ U equilibrium is not affected by the protein content and amyloid self-assembly (Supporting Information).

The T-dependence of the aggregate band intensity at 1618 cm^–1^ is reported in Figure 2c: the signal increases above 40 °C, when a small fraction of U is present (1.4%), in line with the idea that the formation of ordered aggregates requires unfolding. The band reaches a maximum at 54 °C, close to the *T*_m_, then starts decreasing to practically disappear at 80 °C; a characteristic “depletion temperature” *T*_dep_ about 65 °C can be inferred. The disappearance of this band has been already observed in similar T-ranges, when LYS aggregates are formed under reducing conditions^64^ or in the presence of denaturing cosolvents.^72,73^ This depletion indicates the dissociation of the ordered oligomers formed at lower *T* and their rearrangement toward unordered aggregates. In fact, an opaque protein precipitate is observed at the end of the thermal treatment, suggesting that the formation of amorphous aggregates does occur at high *T*. The sample is similar to the opaque particulate gel formed by LYS after incubation at 65 °C and pH = 12 when the low protein charge favors the rapid formation of amorphous aggregates.^34^ We remark that the FTIR AI signal is not sensitive to aggregates of an amorphous nature.^77^ The initial loosening of β-sheet contacts starts at lower temperatures (10 °C or more) than previously observed for analogous structures formed in different environments (presence of DMSO or ethanol and higher pH),^72,73^ suggesting that the thermal stability of amyloid oligomers depends on the solvating conditions. Likely, the high surface charge of lysozyme at pH = 1.8^75^ might induce the formation of relatively small oligomers with a lower thermal stability.

### 3.3 Micro-DSC of Concentrated LYS Solutions

To provide additional insights on the properties of concentrated systems, micro-DSC measurements are performed on the LYS120 and LYS240 samples. Since it is not common to employ this technique for such concentrated samples, an additional more diluted solution of LYS60 is considered to validate the analysis.

**Figure 3:**
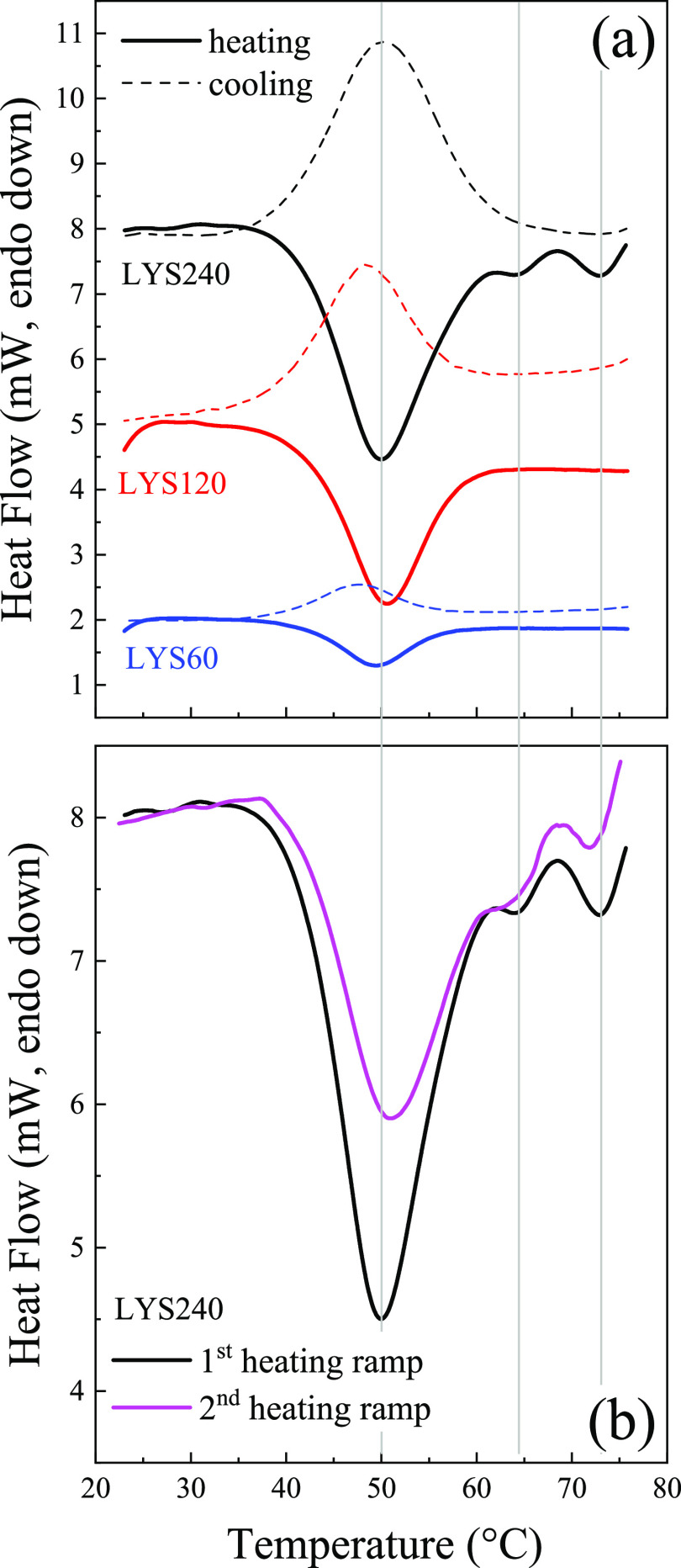
(a) Micro-DSC measures of LYS60, LYS120, and LYS240 solutions, the heating (solid line) and cooling (dashed line) thermograms are shown. The curves are rescaled to a better visualization. (b) Comparison between the first and the second heating ramp for the LYS240 sample.

The calorimetric scans reported in Figure 3a display an endothermic event centered at about 50 °C, consistent with the outcomes of the FTIR results and with the trend reported in literature in regard to the thermal unfolding of LYS at low pH and higher dilution.^42,48,63^ For the LYS60 and LYS120 samples, the profile is typical of a two-state transition with a high degree of reversibility, as assessed by the cooling scans. An apparent enthalpy change of 50 ± 6 kcal/mol (LYS60) and 54 ± 6 kcal/mol (LYS120) can be estimated from the thermograms, in reasonable agreement with the FTIR estimate 67 ± 4 kcal/mol (LYS120), also in view of the simplifying assumptions considered to extract these values. For the sake of comparison, recent DSC investigations on diluted samples led to enthalpy changes of 70^63^ and 100 kcal/mol^48^ at pH = 2, while an average transition enthalpy of 97 kcal/mol (*T*_m_ = 58 °C) results from previous independent studies (LYS concentration from 1 to 10 mg/mL and pH from 2.3 to 2.5).^78^ For the LYS240 sample in which, according to FTIR, ordered aggregates develop, the thermal behavior follows a more complex profile under which several transitions can be identified: after the first peak centered at 50 °C due to unfolding, a transition at 64 °C and a well-defined peak at 72 °C can also be observed. Protein precipitation is found to take place at the end of the heating ramp due to the formation of amorphous aggregates. The formation of opaque irreversible gels has been observed after incubation at 81 °C of more diluted samples at neutral pH.^20^Figure 3a indicates that, even in this case, a significant reversibility of the unfolding process is observed by the cooling scan, suggesting that a large fraction of protein remains in the monomeric form. This is confirmed by looking at the second heating scan reported in Figure 3b. The melting temperature is not affected by the concentration increase and a rather minor reduction of the apparent enthalpy change is observed (Table S4). This is likely due to the interference of amyloid self-aggregation occurring along the melting range. Interestingly, on the basis of a detailed DSC study on quite concentrated LYS solutions (up to 100 mg/mL) at higher pH values, the occurrence of irreversible aggregation, probably of amorphous nature, does not seem to modify very much the features of the unfolding endotherm during the heating cycle.^79^ Overall, DSC results confirm that self-crowding and supramolecular assembly do not alter considerably the F ↔ U equilibrium in these concentrated samples. The second and third endothermic peaks of Figure 3b at 64 and 72 °C are rather small: they might reflect either an entropy driven event (like a dissociation/association phenomenon) or a further conformational change of a partly unfolded state.^64^ Anyhow, the appearance of these features, not evidenced at lower concentrations, should be mainly connected to the development of ordered aggregates that form starting from 40 °C and then tend to disappear at *T* > 55 °C (Figure 2c). The fact that the depletion temperature *T*_dep_ ∼ 65 °C, estimated from FTIR data, is close to that at which the second endothermic feature appears might suggest a common origin. Concerning the peak at 72 °C, in the case of a LYS hydrogel thermally produced upon addition of DTT, an endothermic component found within 75–80 °C was assigned to the melting of gel aggregates.^35^ On the other hand, higher melting points (110–115 °C), assigned to the formation of strong fiber–fiber interaction, are estimated for gels of amyloid fibers.^32^ A different explanation is needed in our case, since amyloid aggregates are not present for *T* > 70 °C. For the LYS240 sample, after the unfolding, several thermal events take place; these include the formation of amyloid oligomers, their dissociation, amorphous aggregation, and other possible conformational changes. As such, a specific attribution to the high temperature features remains elusive.

### 3.4 Kinetics of the Thermal Aggregation and Gelation (LYS240)

**Figure 4:**
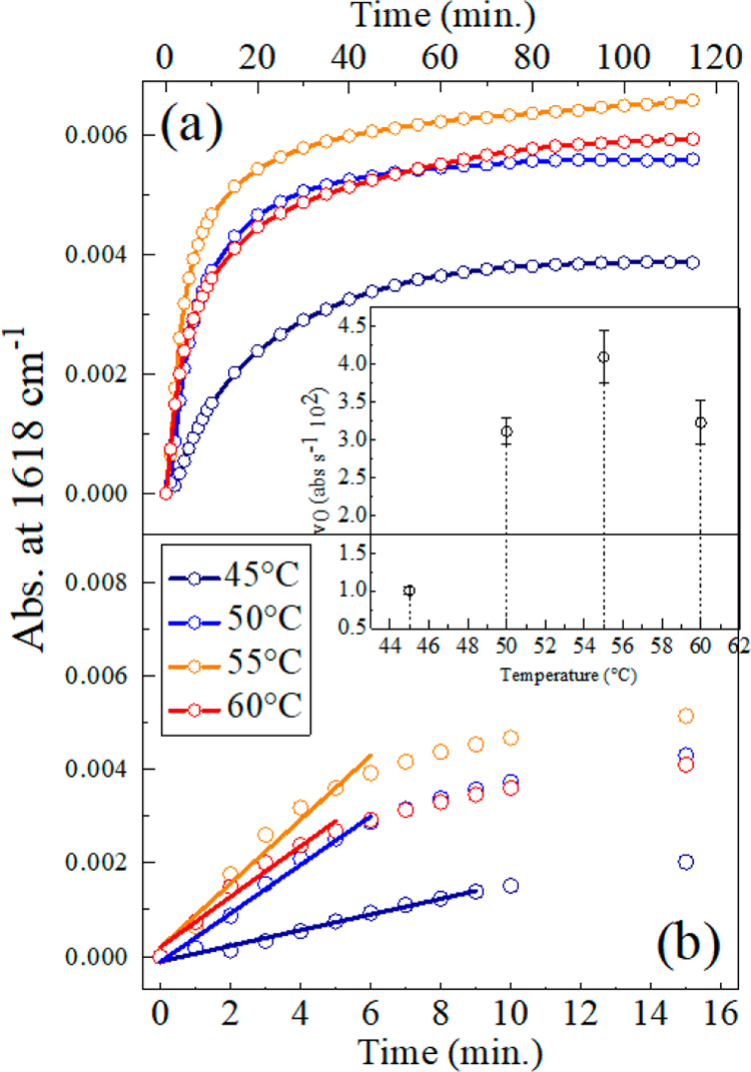
FTIR absorbance of LYS240 at 1618 cm^–1^ as a function of time, determined at different temperatures. Panel (a) shows the evolution obtained up to 120 min, while panel (b) highlights the first 16 min. Straight lines in panel (b) are obtained by a linear curve fitting procedure on the first data point to estimate the initial aggregation rate (*v*_0_). The temperature dependence of *v*_0_ is shown in the inset.

Since the monomer folding/unfolding is relatively fast, in the absence of aggregation, the FTIR spectra (recorded within a few minutes) refer to F ↔ U equilibrium conditions at any *T*. On the other hand, protein aggregation is kinetically controlled, and it could take place at longer temporal scales.^72−74,76,78^ To gain more insight on the aggregation kinetics, FTIR spectra have been collected as a function of time at fixed temperatures. Figure 4 shows the time evolution of the absorbance at 1618 cm^–1^ at four temperatures around *T*_m_; corresponding spectra recorded at 45 and 50 °C are reported in Figure S3.

**Figure 5:**
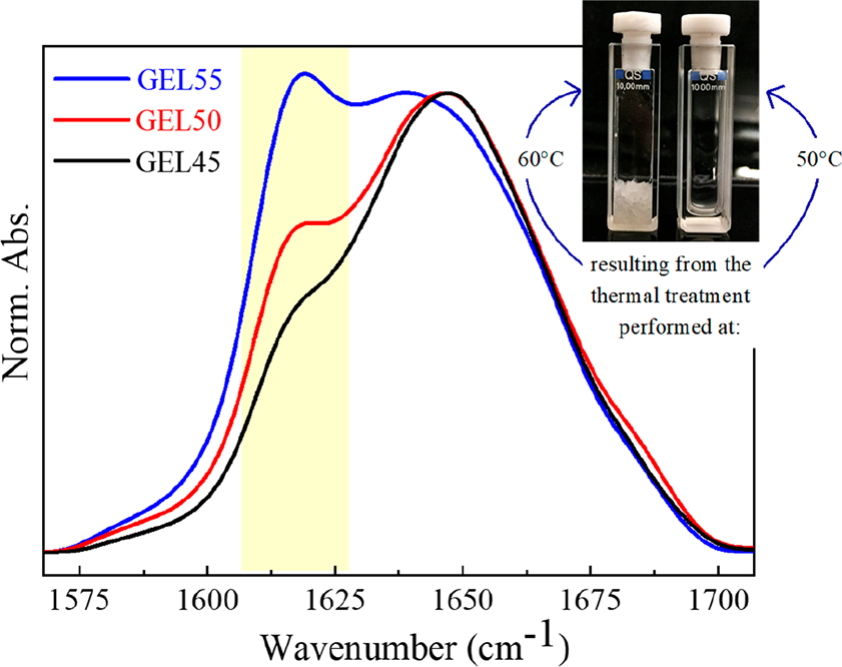
FTIR spectra of GEL45, GEL50, and GEL55 collected at room temperature. The inset shows representative examples of transparent (curing at 50 °C) and opaque (curing at 60 °C) gels.

No aggregate signals are observed below 45 °C, due to the small fraction of U species, and above 60 °C, due to the thermal instability of the ordered oligomers. At intermediate temperatures (Figure 4), the production of the oligomer is fast within the first 10–20 min, then it levels out at longer times. Consistent with the findings reported in Figure 2, the maximum production of aggregates is achieved at 55 °C, which is close to *T*_m_. In addition, the initial aggregation rate *v*_0_, evaluated by a linear fitting of the first data points (Figure 4b), shows a maximum close to *T*_m_ (inset of Figure 5a). The decrease of *v*_0_ observed at 60 °C suggests that the dissociation (or rearrangement) of ordered aggregates starts to be of some relevance at this temperature. At all temperatures, the aggregation rate slows down quite rapidly with time, becoming considerably small after about 30–40 min. Even if the appearance of limiting concentration values (particularly evident for 45 and 50 °C) might reflect a reversible aggregation process,^14^ in the present case, this is attributed to a kinetic arrest, related to the increased number and size of the aggregates. In fact, an increment of the solution viscosity is noticed at the end of the thermal treatment. The reported trends clearly show that, after a 2 h incubation, different (quasi-stationary) fractions of ordered aggregates are produced in the different cases. The subsequent rapid cooling to room *T* of the samples treated at 45 and 50 °C induces the formation of transparent gels (inset of Figure 5) within a few hours. This is often the case also for the sample incubated at 55 °C, even if sometimes a partially opaque gel is observed. Instead, thermal incubation at 60 °C always caused the formation of an opaque gel (inset of Figure 5) already at high temperature, indicating that the amorphous aggregation becomes competitive starting from 55 to 60 °C.

The FTIR spectra of the transparent hydrogels produced after curing at 45, 50, and 55 °C (hereafter referred to as GEL45, GEL50, and GEL55, respectively) are reported in Figure 5 after normalization on the maximum of the band at 1650 cm^–1^. The relative intensity of the band at 1618 cm^–1^, due to the ordered oligomers, increases with the incubation temperature in line with the trends of Figure 4. A visual inspection of the samples indicates that stronger gels are formed at higher temperatures, suggesting a correlation between their mechanical properties and the size and number of the ordered aggregates. Depicted spectra also show that the GEL45 and GEL50 still contain a large amount of α-helix, suggesting that monomers are still present in the jellified samples, in line with the occurrence of a kinetic arrest of the aggregation process. Nevertheless the preservation of residual native-like structures in amyloid oligomers is also expected.^23^ Comparison between the spectra of GEL45 and GEL50 with those of the corresponding viscous liquids (LYS240) recorded at the end of the incubation at 45 and 50 °C (Figure S4), evidenced only minor relative intensity variations of the 1618 cm^–1^ signal. Thus, interprotein β-sheets do not further develop significantly during the gelation process, in which links among ordered aggregates build up a percolating network. The shift of the main band highlights that refolding to the native structure takes place upon cooling; this is mainly ascribed to the dispersed monomers. It can be hypothesized that the transparent gels (GEL45 and GEL50) are made by rather small amyloid oligomers interacting by weak (nonspecific) interactions and contain a fraction of native monomers.

### 3.5 Rheological and Molecular Properties of the LYS Hydrogel (GEL50)

**Figure 6:**
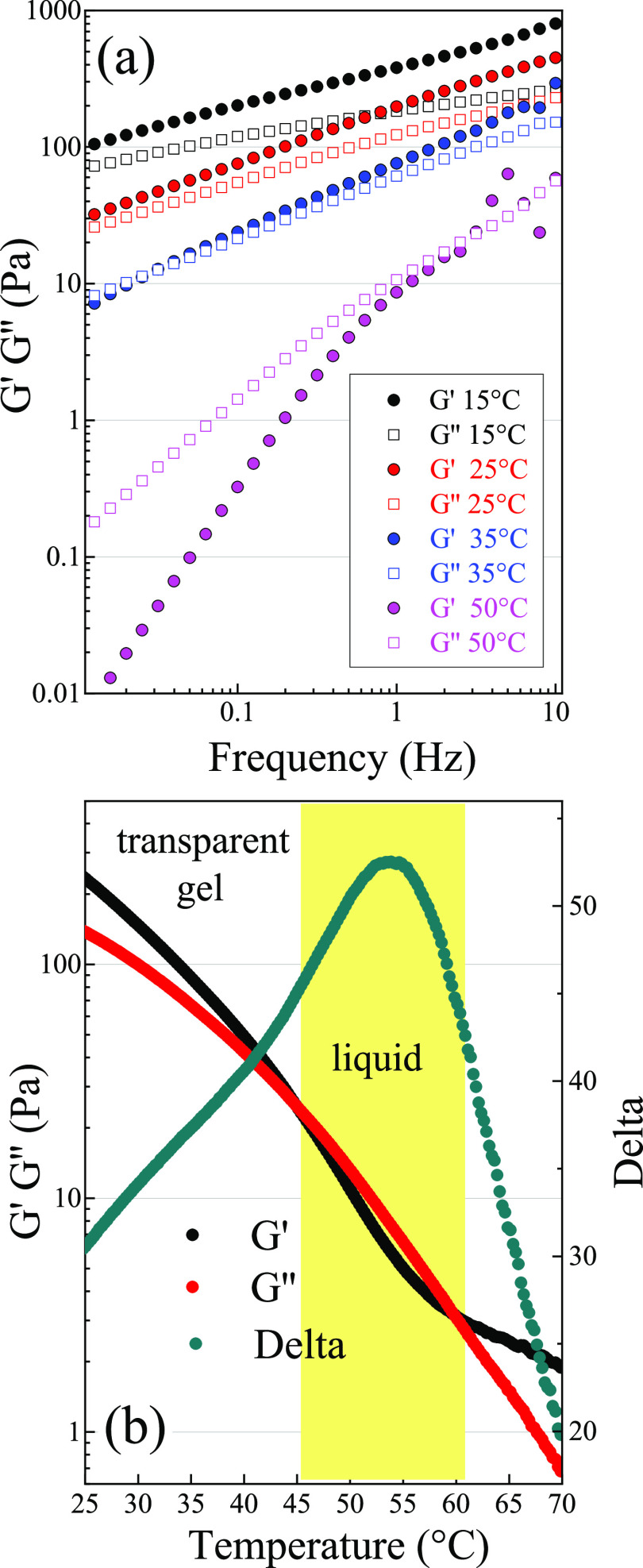
(a) Frequency sweep traces of the GEL50 performed at a shear stress of 0.5 Pa in the frequency range 0.01–10 Hz at different temperatures (15, 25, 35, and 50 °C). (b) Temperature sweep trace of GEL50, conducted at a frequency of 1 Hz and a stress of 0.5 Pa between 15 and 70 °C. The traces of the elastic modulus (*G*′), the viscous modulus (*G*′′), and the phase angle (Delta) are plotted together.

To assess from a mechanical point of view the formation of a real jellified system, a rheological characterization on the transparent GEL50 is performed. Mechanically, a gel is a semisolid system in which the elastic modulus (*G*′) is higher than the viscous modulus (*G*′′) as a function of frequency and temperature.^80^ Otherwise, even if the system does not flow visually after solicitation, it should be considered as a concentrated dispersion and not as a real jellified system. At this extent, frequency sweep tests are performed to investigate the viscoelastic properties of GEL50 at different temperatures, as shown in Figure 6a.

The values of *G*′ and *G*′′ at a given frequency decrease with increasing temperature, reflecting the overall weakening of intermolecular interactions. At 15 and 25 °C, the value of *G*′ is higher than *G*′′ in all ranges of analyzed frequencies. Despite the solid-like behavior that is prevalent (*G*′ > *G*′′), a certain dependence on frequency of the rheological modulus *G*′ is still observable. This feature can be associated with a “weak gel behavior” in which interactions among the chains of the network are not strong, differently from the case of other LYS-based hydrogels.^35^ On the other side, at a higher temperature (from 35 to 50 °C), a crossover frequency between the two moduli appears. This crossover frequency is at around 0.03 Hz at 35 °C, shifting to a higher frequency at 50 °C (∼3 Hz). Thus, this system can be considered as a “weak gel” at 15 and 25 °C, but it behaves as viscoelastic liquid (low consistency dispersion) between 35 and 50 °C. The fact that at low frequency *G*′′ > *G*′ for *T* > 35 °C, highlights the viscous behavior of the system at a longer time scale. This is evidence of the dynamic nature of the gel structure due to the network rearrangements, implying the primary role of the noncovalent bonds in the build up of the gel matrix.^81^ The results obtained from the frequency sweep test are confirmed by temperature sweep test (Figure 6b), highlighting the effect of temperature on the elastic (*G*′) and viscous (*G*′′) moduli of the hydrogel at the frequency of 1 Hz.

At room temperature the sample behaves as a gel, being the elastic modulus (*G*′) higher than the viscous (*G*′′) one (solid-like behavior). As previously evidenced by the sweep test, both moduli decrease with increasing temperature: the material is becoming progressively softer, which can be attributed to the weakening of interactions among aggregates. At 45 °C, some of the links that build up the percolating network are broken. The system is not able to sustain itself anymore and starts to flow (*G*′′ > *G*′ modulus, liquid-like behavior). This transition from the gel to the liquid phase is due to the reduction of the links between the amyloid-like oligomers. Indeed, these are thermally more labile than the cross β-sheet links inside the oligomers themselves, becoming unstable only at *T* > 55–60 °C (Figure 2). This case seems to be different from that of the “strong-link” gels of LYS, produced at room temperature in denaturing conditions, in which interparticle links are more elastic than intraparticle ones.^80^ We also notice that the elastic modulus of the oligomer hydrogel GEL50 is lower by at least 1 order of magnitude with respect to the self-supporting hydrogels developed by Yan et al. (1–4 kPa).^33,35^ However, we recall that their preparation involves the heating up to 85 °C (10 min) of more diluted LYS solutions (∼30–70 mg/mL) in the presence of the reductant DTT and then slow cooling to room conditions. In this way, β-sheet fibrils produced at high temperature further develop during cooling, leading to a fibrillar network through the formation of interfibril junctions.^33,35^ The different stiffness of the GEL50 can then be rationalized based on its different nature, which encompasses the entanglement of small amyloid oligomers rather than more rigid fibrils. This also seems consistent with the findings of Navarra et al., who demonstrated the possibility of forming fibrillar hydrogels of BSA with different strengths after incubation at 60 °C at various pH values: the weakest gel was formed at pH 7.4 when, together with long and thin fibrils, numerous oligomers are also present.^82^Figure 6b shows that above 60 °C the elastic becomes greater than the viscous modulus once again, testifying to a further structural change in the system. In fact, at the end of the thermal treatment an opaque solid-like phase appeared, the formation of which is expected to take place at 60 °C due to oligomer rearrangement and amorphous aggregation, as discussed before.

**Figure 7:**
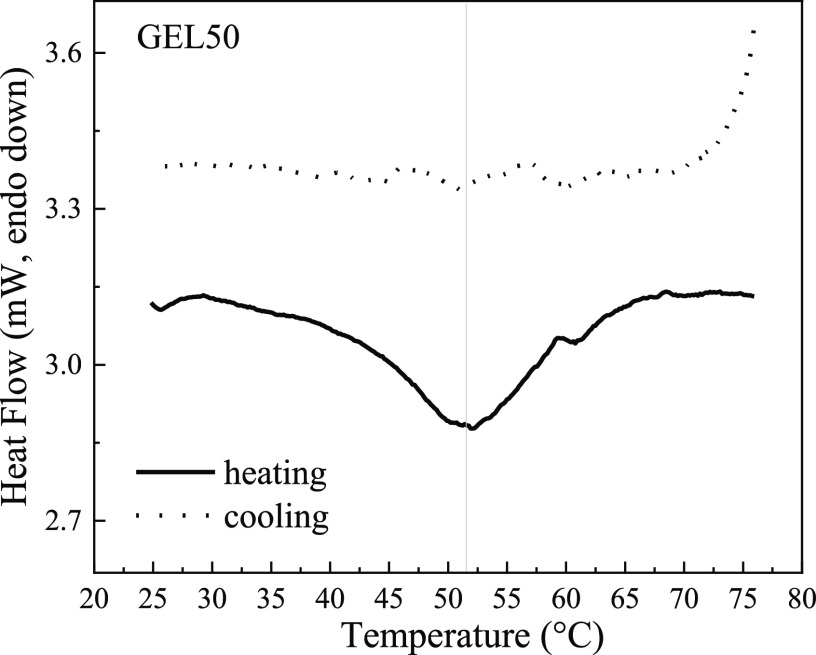
mDSC traces (heating and cooling back) of GEL50.

The DSC trace of this gel (Figure 7) still displays an endothermic transition centered at 52 °C, as previously observed for LYS dispersions, indicating the presence of a fraction of native protein in the hydrogel. Different from the dispersions, the heating of the hydrogel up to 80 °C causes irreversible unfolding of the protein, since no transition is observed during the cooling back scan. From the ratio between the enthalpy associated with the transition of GEL50 and that to the main transition of the LYS240 dispersion, a rough estimate of the fraction of native protein in the hydrogel could be attempted. An approximate value of 12% is obtained as long as interferences of concomitant processes are neglected.

**Figure 8:**
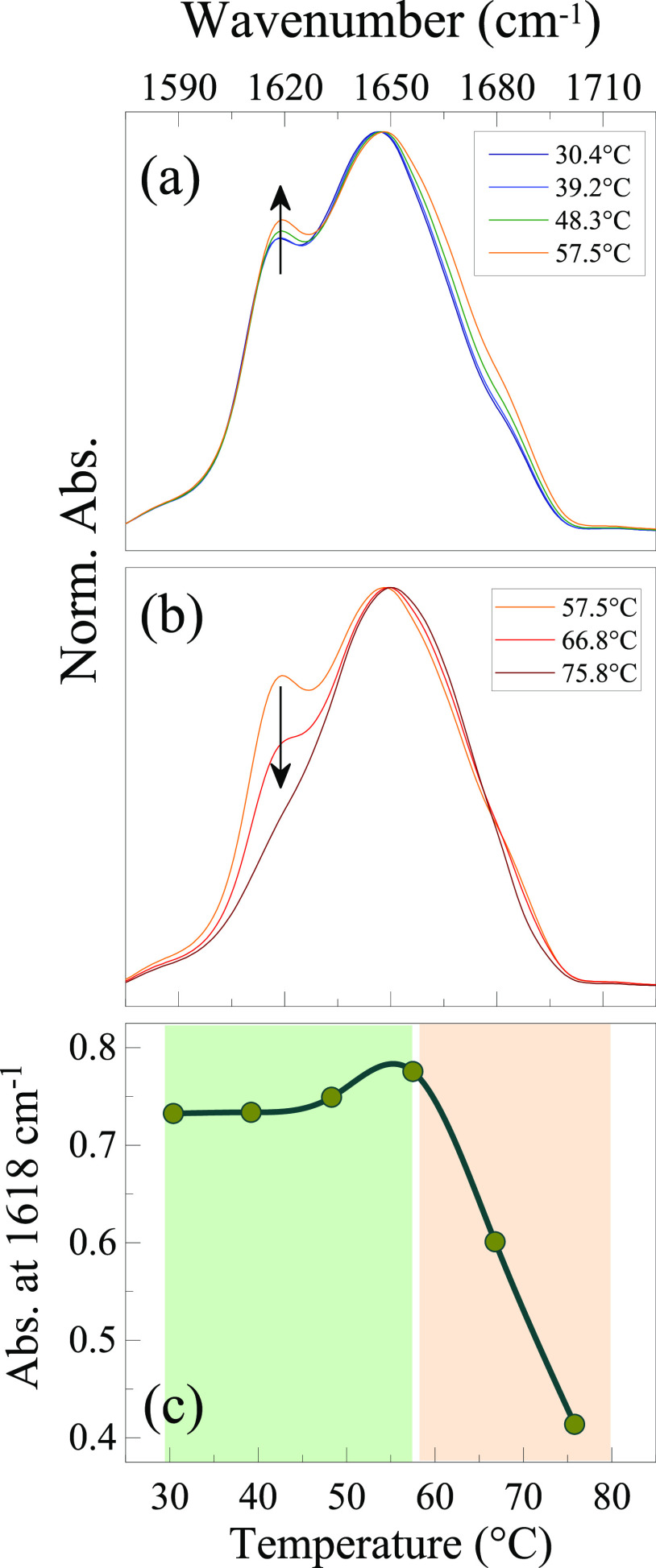
FTIR spectra of GEL50 recorded as a function of temperature. The arrows highlight the increase (a) and decrease (b) of the aggregate signal. The temperature dependence of the absorbance at 1618 cm^–1^ due to ordered aggregates is reported in panel (c).

To explain on molecular terms the rheological and thermal behavior of the hydrogel, FTIR spectra of the same sample are recorded as a function of temperature. Practically no spectral changes are observed going from 30 to 40 °C (Figure 8a,c) when the sample is in the gel phase, confirming that variations of *G*′ and *G*′′ mainly relate to the reduction of (nonspecific) interactions among oligomers, which do not involve the cross β-sheet motifs. As a matter of fact, spectral changes remain rather limited also up to about 60 °C when the gel-to-liquid transition has occurred. The visible blue-shift of the main peak at 1650 cm^–1^ is ascribed to the unfolding of native monomers, still present within the jellified sample, while the small relative increase of the aggregate band observed at *T* > 40 °C (Figure 8a,c ) is due to the restart of oligomers production, consistent with the trends of Figure 5. This clearly means that the gel-to-liquid transition is not connected with the number and size of amyloid oligomers, but rather to the interactions among them. For *T* > 60 °C (Figure 8b,c), the depletion of the band at 1618 cm^–1^ and the shoulder at 1680 cm^–1^ reflects the breaking of the antiparallel β-sheet contacts: in these conditions, the formation of amorphous aggregates is favored.

### 3.6 GEL50 Thermoreversibility and Oligomers Dissociation at High Temperatures

**Figure 9:**
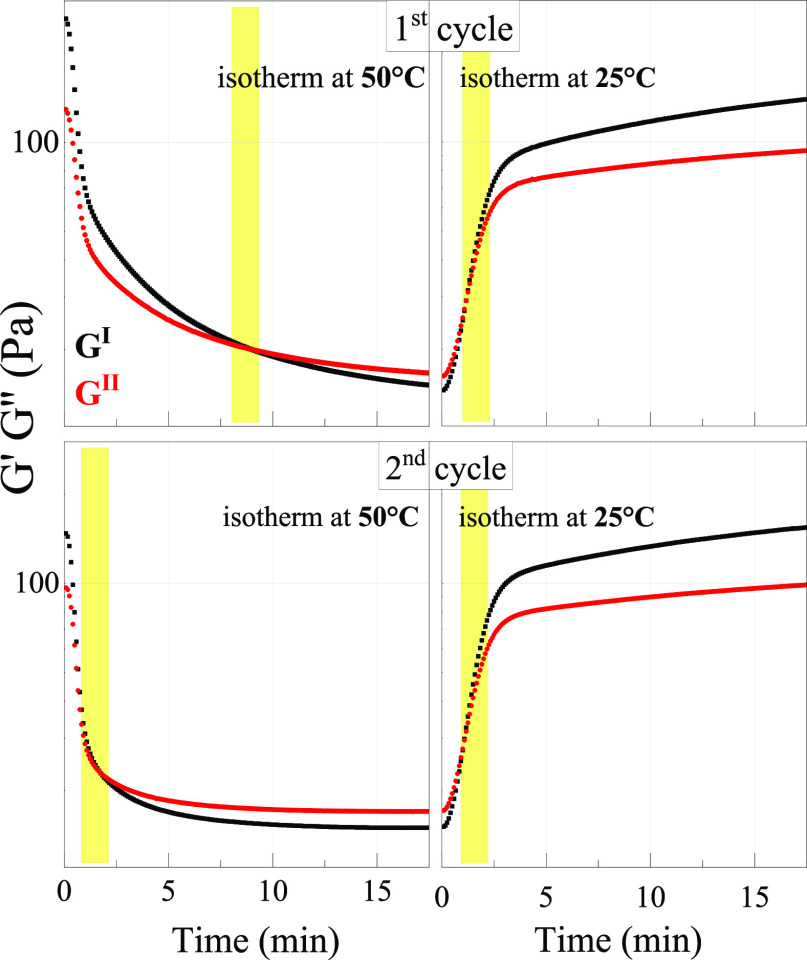
Elastic modulus (*G*′) and viscous modulus (*G*′′) as a function of time. The GEL50 has been incubated at 50 °C (20 min) to follow the gel-to-liquid transition and then cooled back to 25 °C (20 min) to follow the reverse process (first cycle); the same treatment has been repeated on the resulting sample (second cycle).

Figure 9 shows the results of the time sweep test, employed to investigate at selected temperatures the time response of rheological moduli (*G*′ and *G*′′) during the transition from the gel to the liquid states and vice versa. In particular, the elastic and viscous moduli of the hydrogel were measured as a function of time at 50 °C to study the gel-to-liquid transition and then at 25 °C to study the liquid-to-gel reverse process (first cycle); the same analysis was repeated on the resulting sample (second cycle).

During the first cycle the gel-to-liquid transition occurs after about 8 min at 50 °C; this liquid sample reforms a gel when cooled back to 25 °C; in this case, both moduli increase fast within the first 3 min, then their increase slows down. The jellified sample becomes liquid again upon reheating to 50 °C. We notice that now the transition is somewhat anticipated, consistent with the fact that the full stiffness of the gel is not reached at the end of the first cycle due to the limited incubation time. Finally, upon cooling the liquid sample to 25 °C, reformation of the gel is observed again, with a time response analogous to that of the first cycle. Overall, the data indicate that, from a mechanical point of view, this oligomer hydrogel can be considered a thermoreversible one.^33−35^ This capability is strictly related to the possibility of reversibly breaking and reforming the weak links among the aggregates and is an indication of the noncovalent nature of the interactions responsible for the entanglement of the amyloid oligomers during gel formation.^81^ At the same time, the number and size of the amyloid aggregates remain unaltered in the 25 to 50 °C range (Figure 8).

**Figure 10:**
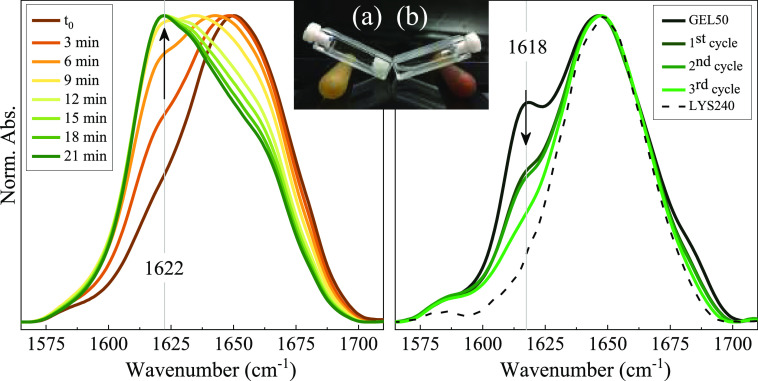
FTIR spectra of the system after the thermal treatments. (a) Long thermal treatment at 80 °C. (b) Short thermal cycles at 80 °C. Inset: the two systems at the end of the thermal treatment.

An attempt has been made to reverse the oligomer formation, exploiting their instability at high temperature. Figure 10a shows the spectra obtained as a function of time for the GEL50 treated at 80 °C. In the initial spectrum (acquisition time about 3 min), the aggregate band at 1618 cm^–1^ is strongly depleted, indicating that amyloid oligomers quickly rearrange at this temperature.

Significant spectral modifications are then detected as a function of time, with an overall intensity redistribution toward lower frequencies. A strong decrease in the relative intensity of the signal at 1650 cm^–1^ is observed, whereas, surprisingly, a new spectral component located at 1622 cm^–1^ grows up, becoming dominant at about a 20 min treatment. If the first effect can be ascribed to the progressive loss of any residual α structure in these drastic conditions, the assignment of the emerged low frequency component is more difficult to understand. Macroscopically, starting from the transparent gel, the sample transforms into an opaque solid-like system (Figure 10a), typically constituted by amorphous aggregates.^18,19,34^ Yet, the presence of a 1622 cm^–1^ component might suggest that, together with amorphous aggregates, also, a fraction of the different amyloid species with a parallel β-sheet structure^44^ and a fibrillary nature were formed.^23^ Their production could be facilitated by hydrolytic and disulfide-bond scrambling^83^ processes that might occur at 80 °C and low pH. Starting from these results, a procedure has been tested, aimed at dissociating amyloid oligomers, characteristic of the transparent gel, to recover the native monomers. The gel was incubated at 80 °C just for 1 min and then quickly cooled down at 25 °C (1st cycle); the procedure has been repeated two other times (2nd and 3rd cycles). The spectrum obtained after each cycle is compared to the spectrum of the initial GEL50 sample and to that of the (H/D exchanged) LYS240 solutions (Figure 10b). As it can be seen, a significant reduction in the 1618 cm^–1^ component is obtained in this way, avoiding other major spectral changes. After the third cycle the spectrum resembles that of the starting solution employed to form the gel, even if the remaining shoulder around 1618 cm^–1^ indicates the persistence of a fraction of aggregates. At the same time, the viscosity of the sample visibly reduces with the number of cycles, and a low viscosity liquid is obtained at the end of the treatment; the sample remains liquid also in the following days. These results indicate that amyloid oligomers of LYS can be dissociated into monomers at high temperatures and that a significant extent of refolding can be achieved by fast cooling. This is necessary to limit other competitive processes such as hydrolysis and formation of other ordered and amorphous aggregates.

## Conclusions

Thermal unfolding and aggregation of highly concentrated lysozyme aqueous solutions (pH = 1.8) are investigated. Formation of protein hydrogels is monitored in situ, and basic structure–property correlations are established.

FTIR experiments show that a two-state model (F ↔ U) is appropriate to describe LYS thermal unfolding at 120 mg/mL, when no aggregation is probed, leading to thermodynamic data (*T*_m_ = 53 °C and Δ*H*_U–F_ ∼ 70 kcal/mol), in line with literature results on diluted solutions. FTIR spectroscopy in conjunction with H/D exchange experiments demonstrate that the thermal stability of LYS does not change upon further self-crowding to 240 mg/mL, even when fast self-aggregation is observed. These findings are supported by DSC experiments. Thus, contrary to some expectations, at low pH values, self-crowding and aggregation only marginally affect the F ↔ U equilibrium.

In the very concentrated sample (240 mg/mL), aggregates with antiparallel β-sheet structures develop at *T* > 40 °C (U percentages larger than ∼1.4%), confirming that the aggregation is triggered by the unfolding. From metastable U species, amyloid aggregates, characterized by antiparallel cross β-sheet links, quickly form in the adopted conditions, reaching a kinetically arrested state. It is envisaged that the self-crowding and the high monomeric charge act together, favoring the rapid formation of (kinetically trapped) amyloid oligomers over that of amorphous aggregates or fibrils. Likely, these oligomers are analogous to those described by Muschol and co-workers, as compact metastable species that might further originate curvilinear fibrils (CFs) or oligomer precipitates (O-Ppt).^25^ From their studies, performed in more diluted conditions, a narrow distribution of oligomeric size was estimated by correlating in situ DLS results with offline calibrated AFM measurements, leading to an average number of 8 monomers per aggregate, which has been modeled as an oblate ellipsoid.^13^ Additional investigations are needed to estimate the specific size and morphology of the amyloid aggregates formed in self-crowded samples, which would be very challenging and beyond the scope of the present study.

Interestingly, our FTIR experiments evidence that amyloid aggregates show a limited thermal stability: their content deceases above ∼55 °C, close to *T*_m_, becoming negligible at 75 °C. Even if aggregation and other rearrangements take place going from 25 to 75 °C, DSC experiments evidence that a large fraction of LYS remains able to refold upon cooling.

Isothermal aggregation kinetics indicate that amyloid species do not form significantly below 45 °C due to the low content of U species and above 60 °C due to their intrinsic thermal instability. Within 45 and 60 °C their formation follows similar trends: the aggregation rate levels off after 20–30 min, leading to quasi-stationary fractions of amyloid structures after a 2 h incubation. The rapid cooling of the samples treated at 45 and 50 °C leads to the formation of transparent hydrogels whose mechanical properties relate to the amount of aggregates produced during the isothermal incubation. Experimental findings strongly suggest that these protein hydrogels are made by amyloid oligomers that build up a percolating network by interacting through rather weak (nonspecific) interactions. A short-range van der Waals attraction and hydrophobic interactions are probably responsible for the interaggregate entanglement.

The amyloid self-assembly is almost arrested upon cooling, which is ascribed to the slowing down of protein diffusion and to the rapid refolding of monomers. In fact, the gel phase still contains a reservoir of native LYS, as confirmed by DSC measurements.

The rheological study of the protein hydrogel (GEL50) confirms its elastic character (*G*′ > *G*′′) at low temperatures, evidencing the occurrence of a gel-to-liquid transition at 45 °C. A comparative FTIR analysis indicates that this transition must be related to the depletion of the weak links among the oligomers. The hydrogel is found to be thermoreversible due to the possibility of breaking and reforming these (nonspecific) interaggregate interactions. The amyloid species themselves can be dissociated back into monomers: a significant degree of refolding is obtained after consecutive cycles, involving short-time exposures at 80 °C (1 min) and fast cooling. Thus, the gel can be retransformed back to a solution of native proteins, supporting the idea that it is constituted by rather small amyloid oligomers.

Overall, results demonstrate that transparent protein hydrogels can be easily formed in self-crowding conditions and their properties can be consistently interpreted considering that they are mainly constituted by amyloid oligomers interconnected by weak (reversible) links. This type of oligomeric hydrogel might be relevant in cellular biology and in the pharmaceutical industry when denaturation occurs in concentrated environments. They could also be considered as a subclass of specific functional biomaterials, along with analogous systems of a fibrillar nature. Containing high quantities of amyloid oligomers, these hydrogels can be further exploited to study the interaggregate entanglement, which must be a rather general process, especially relevant in crowded conditions.

## Supporting Information Available

The unfolding process: a two-state model; Hydrogen–deuterium exchange process; FTIR spectra of LYS240 and gels; Thermodynamic parameters obtained by mDSC (PDF)
