# Supplementary material for: Amyloid Self-Assembly of Lysozyme in Self-Crowded Conditions: The Formation of a Protein Oligomer Hydrogel
Source: Biomacromolecules. 2021 Feb 18;22(3):1147–58. doi: 10.1021/acs.biomac.0c01652 (PMC8023603; doi:10.1021/acs.biomac.0c01652)
Supplement: Supplementary file 1 — bm0c01652_si_001.pdf [file bm0c01652_si_001.pdf]

Supporting Information

Amyloid Self-Assembly of Lysozyme in Self-Crowded Conditions: The Formation of a Protein Oligomer Hydrogel

Sara Catalini,[1] Diego R. Perinelli,[2] Paola Sassi,[3] Lucia Comez,[4] Giovanni F. Palmieri,[5] Assunta Morresi,[3] Giulia Bonacucina,[2] Paolo Foggi,[1,3,6] Stefania Pucciarelli,[7] and Marco Paolantoni [3,]*

[1] *European Laboratory for Non-Linear Spectroscopy (LENS), University of Florence, 50019 Sesto Fiorentino, Italy*

[2] *School of Pharmacy, University of Camerino, 62032 Camerino, Italy*

[3] *Department of Chemistry, Biology and Biotechnology, University of Perugia, 06123 Perugia, Italy*

[4] *IOM-CNR c/o Department of Physics and Geology, University of Perugia, 060123 Perugia, Italy*

[5] *School of Pharmacy, University of Camerino, 62032 Camerino, Italy*

[6] *National Metrological Research Institute (INRIM), Strada delle Cacce 91, 10135 Torino, Italy*

[7] *School of Biosciences and Veterinary Medicine, University of Camerino, 62032 Camerino, Italy*

*****Corresponding author: marco.paolantoni@unipg.it

1) The unfolding process: a two-state model

The unfolding process can be described by a two-state model, based on the equilibrium between folded (F) and unfolded (U) species (F↔ U) [1,2].

The spectroscopic observable y_T (band position or signal intensity) can be expressed as the sum of its value in the folded and in the unfolded states weighted for their corresponding population. Considering a linear temperature dependence of the observable within each of the states leads to the following expression:

$$y_T = \frac{y_F + m_F \cdot T + (y_U + m_U \cdot T) \cdot K_T}{1 + K_T} \qquad \text{S1}$$

K_T is the equilibrium constant at a given temperature, y_F and y_U are the values of the observable when the proteins are respectively in the folded and in the unfolded state at T= 0 K, m_F and m_U describe their temperature dependence in pre and post-melting region.

The equilibrium constant can be written as a function of the unfolding enthalpy (ΔH_{F-U}) and of the melting temperature (T_m):

$$\ln(K_T) = \frac{\Delta H_{F-U}}{R} \cdot \left(\frac{1}{T_m} - \frac{1}{T}\right) \qquad \text{S2}$$

R is the gas constant.

From the two relationships, the following one can be obtained:

$$y_T = \frac{y_F + m_F \cdot T + (y_U + m_U \cdot T) \cdot e^{\frac{\Delta H_{F-U}}{R} \cdot \left(\frac{1}{T_m} - \frac{1}{T}\right)}}{1 + e^{\frac{\Delta H_{F-U}}{R} \cdot \left(\frac{1}{T_m} - \frac{1}{T}\right)}} \qquad \text{S3}$$

This functional form was employed to reproduce the melting curves.

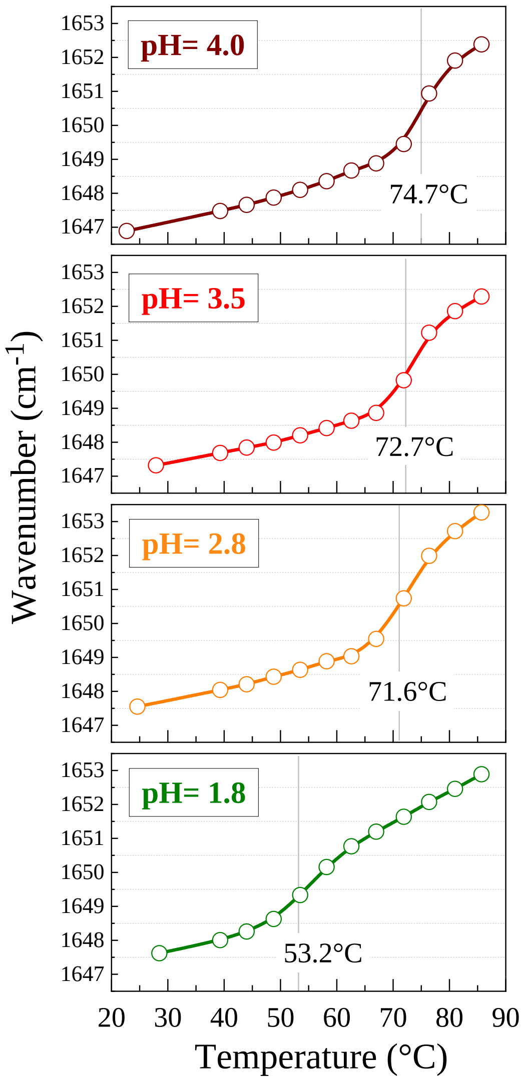

Figure S1: AI frequency shift of LYS120 solution at different pH, as a function of temperature.

**pH**	$\mathbf{T_m}$**(°C)**	$\mathbf{\Delta H_{F-U}}$**(kcal mol^{-1})**
4.0	74.7 $\pm$ 0.9	113 $\pm$ 20
3.5	72.7 $\pm$ 0.5	116 $\pm$ 15
2.8	71.6 $\pm$ 0.6	85 $\pm$ 8
1.8	53.2 $\pm$ 0.3	67 $\pm$ 4

Table S1: Thermodynamic parameters obtained by reproducing the experimental data (AI peak position) with the two-state model function.

2) Hydrogen-Deuterium exchange process

To describe H/D exchange kinetic in proteins it is possible to adopt the following model [3,4]:

$$\mathrm{F(H)} \underset{k_{-1}}{\overset{k_1}{\rightleftarrows}} \mathrm{U\,(H)} \xrightarrow{k_s} \mathrm{U(D)} \qquad \text{S4}$$

F(H) and U(H) are the hydrogenated folded and unfolded species, U(D) the unfolded deuterated one. k_1 and k_{-1} are the kinetic constant of unfolding and refolding, respectively, while k_s is the kinetic exchange constant for the protein in the unfolded state. The collective exchange pseudo constant might be written as:

$$k_{ex} = \frac{k_1 k_s}{k_{-1} + k_s} \qquad \text{S5}$$

If $k_s \gg k_{-1}$, the hydrogen exchange constant is much greater than the refolding one and the experimental constant equals the unfolding one:

$$k_{ex} = k_1 \qquad \text{S6}$$

If $k_{-1} \gg k_2$, the refolding constant is much greater than the isotopic exchange one and the experimental constant is given by:

$$k_{ex} = K_T\, k_s \qquad \text{S7}$$

In this limit the protein unfolds and refolds many times before that all the exchangeable hydrogens of the segment can be replaced by deuterium.

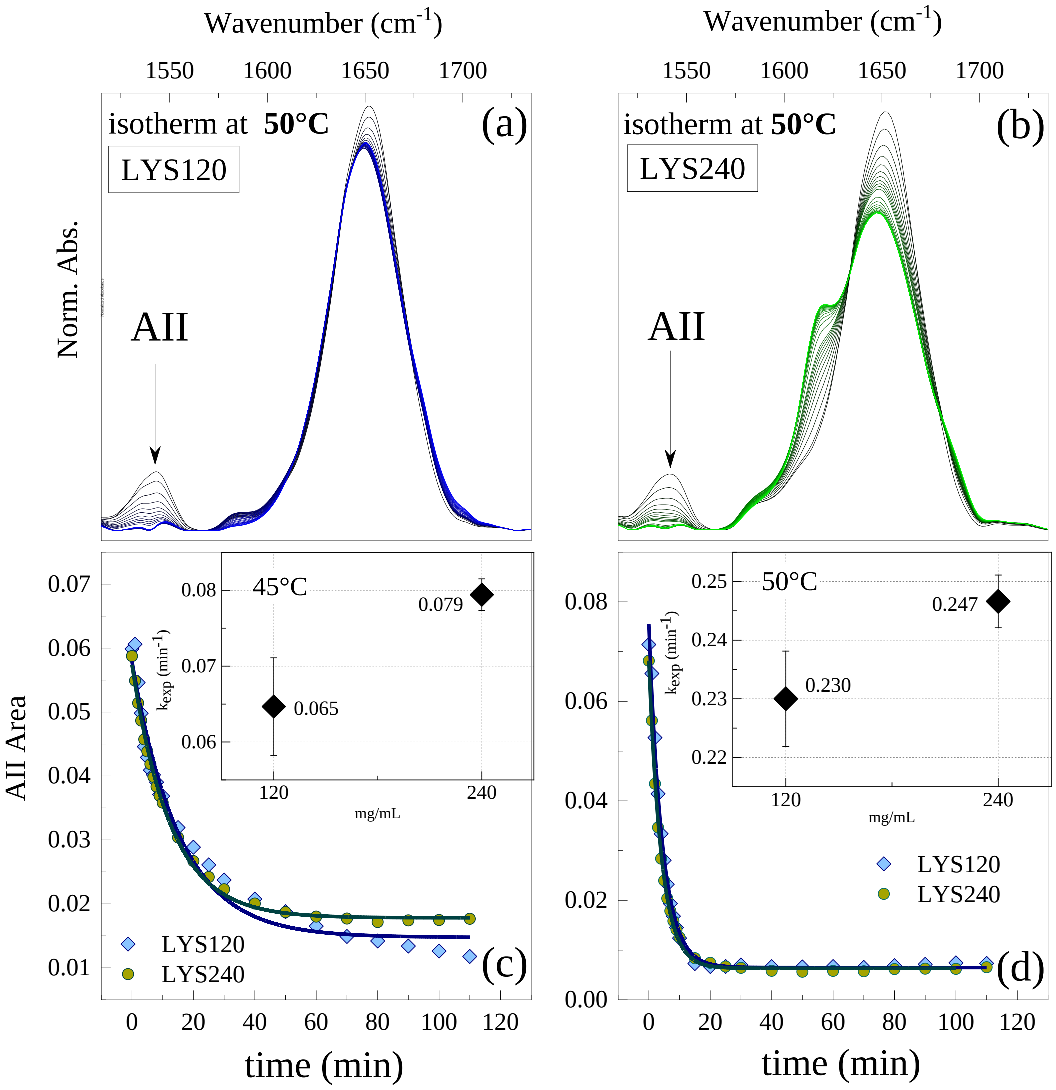

Figure S2: FTIR spectra of LYS120 (panel a) and LYS240 (panel b solutions recorded at 50°C as a function of time. Panel (c) and (d) report the area values of AII band at 45 and 50°C respectively. The experimental data are fitted using an exponential function, whose inverse value is the experimental exchange kinetic constant ($k_{ex} = \frac{1}{\tau}$).

**[LYS]** (mg/mL)	**T** (°C)	**K_T**	**k_{ex}** (min^{-1})
120	45	0.07	0.065 ± 0.006
240			0.079 ± 0.002
120	50	0.36	0.230 ± 0.008
240			0.247 ± 0.005

Table S2: Equilibrium constant (K_T) and experimental exchange kinetic constant (k_{ex}) values at different experimental conditions. Error bars only account for the uncertainties derived from the fitting procedures.

The increase of k_{ex} with temperature can be mainly ascribed to the increased fraction of U species, in line with Eq. S7, even if, likely, the limit expressed by this equation is not fully reached here. Overall, the data confirm that the exchange process is only marginally affected by the protein concentration.

3) FTIR spectra of LYS240 and gels

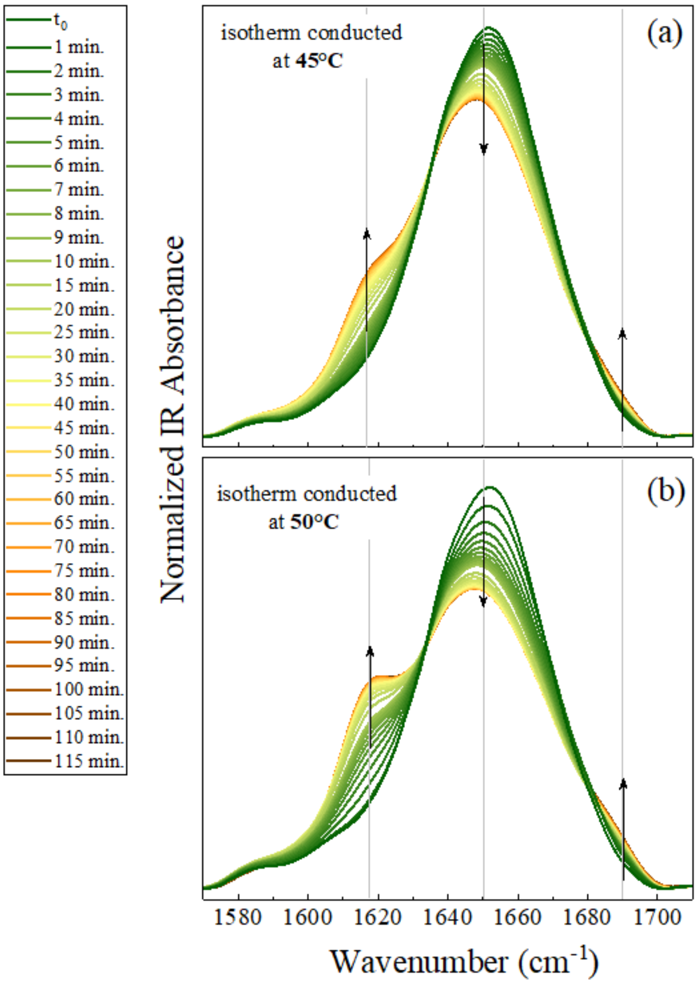

Figure S3: FTIR spectra of LYS240 recorded at 45°C (panel a) and 50°C (panel b) as a function of time.

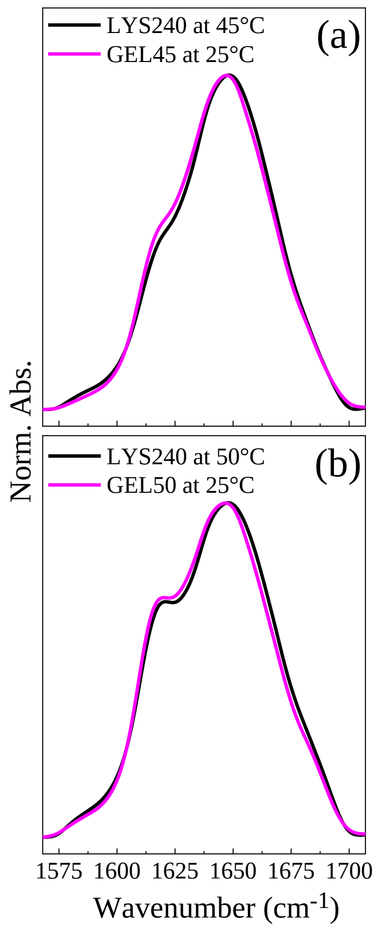

Figure S4: comparison between the spectra of LYS240 after two-hours of thermal incubation at 45 (a) and 50 °C (b) and the corresponding GEL45 (a) and GEL50 (b) obtained upon cooling.

4) Thermodynamic parameters obtained by mDSC

**Samples**	**T_m(°C)**	**ΔH_{F-U} (kcalmol^{-1})**
LYS60	49 ± 2	50 ± 6
LYS120	50 ± 2	54 ± 6
LYS240	50 ± 2	45 ± 5
GEL50	52 ± 2	5.3 ± 0.3

Table S4: Melting temperature (T_m) and enthalpy change (ΔH_{F-U}) obtained by mDSC.

References

1. Sassi, P.; Giugliarelli, A.; Paolantoni, M.; Morresi, A.; Onori, G. Unfolding and aggregation of lysozyme: A thermodynamic and kinetic study by FTIR spectroscopy. *Biophys. Chem.* **2011**, 158(1), 46-53.
2. Jackson, S. E. How do single-domain proteins fold? *Fold. Des.* **1998**, 3(4), R81-R91.
3. Smith, D. L.; Deng, Y.; Zhang, Z. Probing the non-covalent structure of proteins by amide hydrogen exchange and mass spectrometry. *J. Mass Spectrom.* **1997**, 32(2), 135-146.
4. Klotz, I. M.; Frank, B. H. Catalysis by imidazole of deuterium-hydrogen exchange in amide NH groups. *Science* **1962**, 138(3542), 830-831.
